# Community engagement and pediatric obesity: Incorporating social determinants of health into treatment

**DOI:** 10.1017/cts.2019.447

**Published:** 2019-12-19

**Authors:** Joseph A. Skelton, Deepak Palakshappa, Justin B. Moore, Megan B. Irby, Kimberly Montez, Scott D. Rhodes

**Affiliations:** 1 Department of Pediatrics, Wake Forest School of Medicine, Winston-Salem, NC, USA; 2 Brenner FIT (Families in Training) Program, Brenner Children’s Hospital, Winston-Salem, NC, USA; 3 Program in Community Engagement, Wake Forest School of Medicine, Clinical and Translational Science Institute, Winston-Salem, NC, USA; 4 Department of Internal Medicine, Wake Forest School of Medicine, Winston-Salem, NC, USA; 5 Department of Family and Community Medicine, Wake Forest School of Medicine, Winston-Salem, NC, USA; 6 Maya Angelou Center for Health Equity, Wake Forest School of Medicine, Winston-Salem, NC, USA; 7 Department of Social Science and Health Policy, Division of Public Health Sciences, Wake Forest School of Medicine, Winston-Salem, NC, USA

**Keywords:** Obesity, pediatric, community, engagement, treatment

## Abstract

Childhood obesity is a complex and multi-faceted problem, with contributors ranging from individual health behaviors to public policy. For clinicians who treat pediatric obesity, environmental factors that impact this condition in a child or family can be difficult to address in a clinical setting. Community-clinic partnerships are one method to address places and policies that influence a person’s weight and health; however, such partnerships are typically geared toward community-located health behavior change rather than the deeper social determinants of health (SDH), limiting effective behavioral change. Community-engaged research offers a framework for developing community-clinic partnerships to address SDH germane to obesity treatment. In this paper, we discuss the relationship between SDH and pediatric obesity treatment, use of community-clinic partnerships to address SDH in obesity treatment, and how community engagement can be a framework for creating and harnessing these partnerships. We present examples of programs begun by one pediatric obesity clinic using community-engagement principles to address obesity.

## Introduction

Over a third of children in the US have overweight or obese [1], and increasing numbers of children are now classified as having severely obese [2]. Causes are multifactorial, with dietary changes (e.g., increasing exposure to energy-dense fast food, consumption of sugar-sweetened beverages) and environmental modifications (e.g., increased mechanized transport, sedentary activities) as the leading culprits. Many health behaviors are influenced by “upstream” social and economic factors such as health literacy and education, economic opportunities, and health care [3].Unfortunately, these can only be addressed through policy changes that are difficult to enact, costly, and may take a generation to produce observable health outcomes. Thus, how can health care providers treat children with obesity and support their families when socioeconomic contributors are currently beyond their scope of practice?

The etiologies of pediatric obesity are complex, and its prevention and treatment must account for social and economic contributors. Effective treatment and access to resources are vital for children who are at high risk of persistent obesity as adults with concomitant health problems [4]. Children from disadvantaged and under-resourced backgrounds have the highest prevalence and risk of obesity [2]. These deeper, socioeconomic contributors are typically referred to as social determinants of health (SDH), succinctly described as “the conditions in which people are born, grow, work, live, and age, and the wider set of forces and systems shaping the conditions of daily life” [5]. Healthy People 2020 (Office of Disease Prevention and Health Promotion) conceptualizes SDH into five constructs: economic stability, education, social and community context, health and health care, and the neighborhood and built environment. (https://www.healthypeople.gov/2020/topics-objectives/topic/social-determinants-of-health). While these factors influence the development of obesity and prevent sustainable behavior change [6], approaches to addressing these factors are not always well defined, falling under the realm of “social work,” or are limited by barriers of clinic-based interventions [7]. As such, SDH has fallen under the domain of public health, community organizations, and social service agencies, often detached from clinical settings [8,9]. However, without addressing environmental and societal contributors to obesity, such as food availability and access to care, clinical treatment programs and other types of behavioral change interventions likely have limited effectiveness [6].

Community engagement [10], as a research approach, offers a framework for developing community-clinic partnerships to address the SDH affecting obesity treatment. In this paper, we discuss the impact of SDH on pediatric obesity treatment, and how community engagement can be a framework for creating and sustaining these partnerships. We seek to explore the strategies of community-engaged research applied by our community-clinic partnerships to address child obesity through an SDH lens. We provide an example of one pediatric obesity clinic using community-engagement principles to address obesity treatment.

### SDH and Pediatric Obesity Treatment

The United States Preventive Services Task Force (USPSTF) recommends comprehensive, intensive, behavioral treatment to address childhood obesity [11]. The best evidence suggests that 26+ hours of contact between a patient, family, and treatment program, with a focus on family health behavioral (versus individual child) interventions, is needed to elicit improvement in a child’s weight status [11]. Changes outside such programs are problematic in families from low-socioeconomic brackets and those living in under-resourced neighborhoods. Challenges include food deserts and swamps [12]; lack of physical activity opportunities, such as safe, walkable neighborhoods [13]; and access to parks and other avenues for physical activity [14]. Treatment approaches can focus on SDH as well as health behaviors, which influence the health and weight of individuals. For clinical obesity programs, clinicians can engage with community organizations to address challenges inhibiting healthy behavior change.

### Community-clinic Partnerships to Address Pediatric Obesity

Interest in community-clinic partnerships is increasing. For example, Taveras et al. are studying a community-clinic partnership that includes health coaches working in the community; initial results are favorable [15,16]. Furthermore, in line with USPSTF recommendations [11], Hoffman et al. increased treatment contact hours by partnering with a local parks and recreation department to deliver physical activity interventions to children in an obesity program [17]. In addition to demonstrating feasibility, they increased physical activity and improved quality of life (without significant changes in weight status in this pilot). These studies demonstrate the potential of clinical-community partnerships to address pediatric obesity.

Consensus [18,19] and evidence [16,17] suggest community-clinic partnerships are necessary to address the increasing number of children with obesity, since independently each approach appears to be insufficient. The behavior change that clinicians hope to elicit “outside the clinic” necessitates engaging the community to support families attempting change. Some SDHs (e.g., income, neighborhood safety, and food deserts/swamps) fall into the realm of policy, while others (e.g., access to care, activity resources, and food insecurity) are more amenable to a partnership approach. We describe real-world examples from our clinic that may guide professionals and lay leaders in developing effective community-clinic partnerships.

### Community Engagement as a Framework

One guiding framework for a community-clinic partnership to address SDH impacting obesity is community-engaged research, a “process of working collaboratively with groups of people affiliated by geographic proximity, special interests, or similar situations with respect to issues affecting their well-being” [10]. Community engagement requires clinicians to develop an authentic relationship with community members and organizations. Clinicians should recognize the diversity and strengths of community organizations, including representation of a particular race or ethnic group, identification with a defined geographic area or neighborhood, and targeted population (e.g., families struggling with obesity). Providers may also be diverse, representing areas of clinical expertise from dieticians to physicians.

Community engagement must feature respect for all partners, with mutual benefit at the forefront of collaboration. Therefore, it is imperative for all involved to know and internalize the stated goals of community engagement: enlist new resources and allies; build trust; create better communication; and improve overall health outcomes as projects evolve to lasting partnerships [10]. Here, we describe the approach of one multidisciplinary pediatric obesity treatment program that uses community-engaged research processes to overcome barriers to treatment, address SDH, and increase clinical and community capacity for addressing obesity.

### Brenner FIT®

Brenner FIT® (Families In Training) is a pediatric obesity treatment, prevention, research, and education program in Winston-Salem, North Carolina, founded in 2007 [20]. It is part of Brenner Children’s Hospital, the pediatric arm of Wake Forest Baptist Medical Center, an integrated academic healthcare network. The core mission of Brenner FIT is to provide evidence-based weight management for children ages 2–18 years with their family. Any physician or advanced practice provider can refer patients across a 19-county catchment area of northwest North Carolina. Although the child’s entire family is welcome to attend each clinic visit, at least one parent or primary caregiver must attend all visits with the child. Families attend monthly clinic visits and periodic group education classes for 6 months. Family centered treatment uses motivational interviewing [21], focusing on nutritional and activity behaviors of the family. Group classes employ an experiential education approach to learning [22], providing hands-on opportunities for children and families, including cooking classes, activity programming, and parent role play. We have published evidence of effectiveness of these approaches within our diverse population [23–25].

### Community Engagement and Brenner FIT®

Pediatric obesity treatment is focused on family based education (versus child-based), behavior modification (record keeping, stimulus control, and goal setting), nutrition and activity counseling (culturally effective and developmentally appropriate), and assessment and management of associated health concerns [20]. Evidence-based adult weight management typically involves approaches inapplicable for youth (e.g., calorie counting, limited portion sizes, or developmentally inappropriate activity). Safe, family based, age-appropriate resources may be scarce among accessible community-based resources.

The association between poverty and obesity means those most affected by obesity have additional challenges and stressors that complicate patients’ ability to make health behavior changes. Furthermore, community wellness resources are less able to address key variables such as food insecurity, stress, and transportation. The following are examples of how Brenner FIT uses community-engaged approaches to support well-being of children and families presenting for weight management.

### SDH and Brenner FIT Experiences

#### Access to care

Over 20% of families in Brenner FIT only speak Spanish in their home (Table 1) [24]. The Latinx population in the Brenner FIT catchment area includes authorized and unauthorized persons. Brenner FIT counselors screen parent and children for mental health issues, which have been linked to childhood obesity [26] and can impede outcomes [27]. Children complete the Pediatric Symptom Checklist [28] and are referred for evaluation if they score above a certain cutoff; parents are asked if they previously or presently have concerns about: mental or behavioral health, substance use, stress in regard to relationships and parenting, support systems, or domestic violence. Mental health concerns are frequently identified during initial assessment, most commonly parental depression and anxiety [29,30]. In the central piedmont region of North Carolina, mental health practitioners typically have private solo practices or independent community-based practices. Few Spanish-language mental health providers accept Medicaid insurance (for the child) or provide discounted services (for parents without insurance). Thus, Brenner FIT staff undertook a networking approach to identify potential provider partners. They then contacted these mental health providers to establish relationships, build lines of referral and communications, and understand their respective missions, as well as provided means in which to contact Brenner FIT. This collaboration has connected Brenner FIT patients and parents with mental health providers and provided a reliable and confidential resource for Spanish-speaking patients.

**Table 1.** Brenner FIT and social determinants of health (SDH) community engagement

SDH domain	Community-engagement result
Access to care	- Developed relationships with bilingual mental health providers
	- Swimming and bicycling classes
Food insecurity	- With partners, organized emergency food aid for patients and families
	- Educational series for families with food insecurity
	- Established process to screen for food insecurity, then provide referrals and resources
Transportation	- Vouchers for travel to clinic
	- Telemedicine program
	- Developed lay-led community-based program for rural areas
Health behaviors	- Community-based teaching kitchen
	- Volunteer training program
	- Mobile teaching kitchen
Parenting education and support	- Community-based parenting classes

Many children in Brenner FIT come from underserved and under-resourced neighborhoods, often lacking access to physical activity. In clinical interactions, many parents and children mentioned an interest in biking and swimming, but their children not knowing how to do either. While anecdotal, this was a frequent topic of discussion. A local group offered free swim lessons, and so far, over 20 children have received swim lessons. Similarly, Brenner FIT partnered with a non-profit volunteer organization that distributes free helmets and teaches children how to safely ride bicycles. Two to three times yearly, the group holds events for patients and families of Brenner FIT to teach children how to ride bicycles. This collaboration has also connected local volunteers who repair and refurbish used bicycles to donate to families. The programs are purposefully held in the evenings or weekends, which aid in families being able to attend without missing work or school, and transportation is less of a struggle (locations are on public transportation route, working parents can attend and bring child). When appropriate, we have provided transportation vouchers to families or connected them with other services to assist them. While “non-traditional” by obesity treatment standards, these programs have been a positive partnership between Brenner FIT and community-based organizations. After each session, discussions were had between the swimming and biking programs to determine changes to make in future events and give bi-directional feedback.

#### Food insecurity

Increased hunger and food insecurity predict higher obesity risk [31,32]. In one obesity program, 24% of participants screened positive for food insecurity [33] but few families had completed enrollment for food assistance. Brenner FIT connects and meets in-person with several hunger relief organizations regularly to build relationships and facilitate patient referrals. Examples of community organizations addressing hunger in their community include a for-profit community-sponsored agriculture (CSA) company; a non-profit women’s shelter with consistent excess of donated food items; a church program that placed post-graduate student interns completing a year of service in the community; and a national grocery store chain with a store in a predominantly minority-background neighborhood. The CSA donated excess produce for families in Brenner FIT, which then provided guidance in vegetable preparation. The grocery store delivers food basket items free-of-charge to the food pantry of the Brenner FIT primary care partner, which resulted in significant cost and time savings. The church program placed an intern with Brenner FIT to facilitating administrative processes for obtaining and storing donated food items, ordering and tracking food distribution in the food pantry, and developing an educational series for patients struggling with food insecurity. All these partnerships have continued to develop and expand, with regular meetings and communications, and periodic recognition of the efforts of the community partners.

#### Transportation

Obesity treatment involves frequent clinical appointments [11], and lack of regular or reliable transportation causes missed visits [34] and increased attrition [35]. To address this challenge, Brenner FIT built upon an existing relationship with a primary care partner (an urban, non-profit health center affiliated with the medical center) which provides transportation vouchers for patients. Families whose medical home is this health center (over 30% of Brenner FIT patients) may receive transportation vouchers to attend Brenner FIT clinic visits and educational programs. Establishing a telemedicine network has also mitigated access issues [23]. This involved placing a secure telemonitor in a local health department, a satellite clinic of the hospital, or primary care offices in outlying areas. These partnerships led to discovering other community resources and local partners, such as local YMCAs, food resources, and mental health providers.

One such collaboration with the health department resulted in a grant application to develop and deliver a community-based program for families concerned about their child’s weight. Through this collaboration, Brenner FIT developed on-line training modules for health department employees, who serve as lay-health advisors and lead monthly classes on ways to improve family health through behavior change and family activity. Brenner FIT clinicians provide ongoing support and training for these lay leaders. Part of this was a continuing education symposium that was free for community partners, which included opportunities for them to provide feedback to Brenner FIT.

#### Health behaviors

**Fig. 1.**
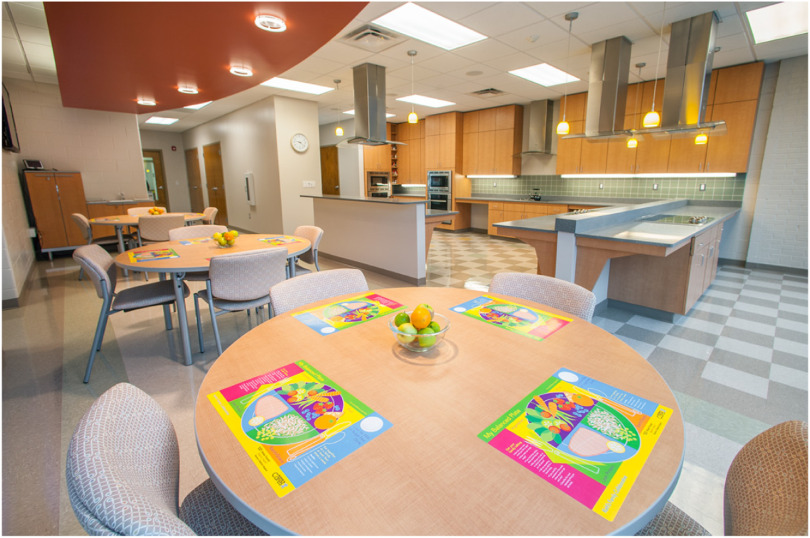
Teaching kitchen.

Increasing the number of family meals cooked at home is an important goal in treating pediatric obesity [36]. Based on clinical experience and requests from participating parents, Brenner FIT and a local YMCA partnered to build a teaching kitchen in their community location (Fig. 1). In addition to hundreds of classes led by Brenner FIT, a training program now prepares volunteers to lead family based cooking programs. YMCA staff are trained to deliver classes to community members, employing the Brenner FIT teaching kitchen and its recipes and educational materials. This partnership has expanded to include a summer camp program, pre-school curriculum, and staff wellness activities. Additional volunteer organizations are being trained to lead nutrition and culinary education programs open to community members. Quality improvement projects have demonstrated to us a high level of satisfaction by parents, and they report continuing to cook recipes learned in classes.

**Fig. 2.**
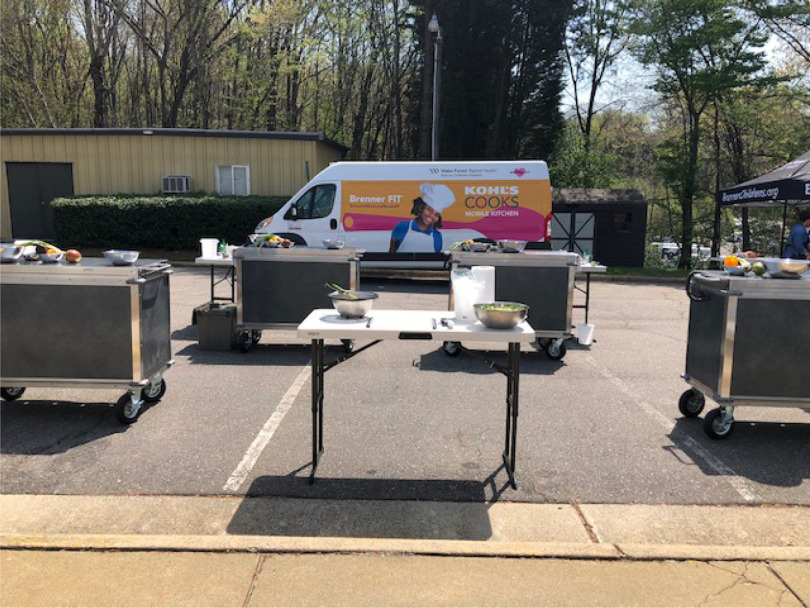
Mobile teaching kitchen.

Based on work in food insecurity, a working relationship was established with a hunger organization that has a culinary job training program. Their director and executive chef provided key assistance in designing a mobile food kitchen (Fig. 2), which subsequent grant funding has supported. This cargo van transports equipment to community locales for cooking classes. Two to three times a month, Brenner FIT leads a cooking class with community partners at their site. The mobile kitchen was a direct response to the community’s request for more culinary resources in their neighborhoods.

#### Parenting education and support

Parenting practices and child obesity are inextricably connected [37]. Building on an established research relationship, a local Latinx service organization requested Brenner FIT’s assistance in providing parenting support. Brenner FIT staff members, trained in a parenting support program, began providing parenting classes at the community partner’s location. This arrangement enables Brenner FIT to link parents of their patients with the community organization when they need services and support.

The efforts and programs above have associated costs, presenting challenges to their sustainability. Several approaches have been used to ensure ongoing services. For the non-profit organizations (swimming lessons, bicycle lessons, and food insecurity organizations), their work with Brenner FIT is included in their yearly fundraising, or joint fundraising events are held. Media reach and recognition of these partnerships is pursued jointly by the hospital system and the community organizations, increasing philanthropic reach. Other organizations considered it a part of their yearly philanthropic or in-kind community outreach. The teaching kitchen and mobile kitchen were funded through development grants to local and national organizations; this provided initial, one-time costs, and some ongoing expenditures with invitations to apply yearly for renewal grants. North Carolina has pursued a Medicaid waiver, which would provide funds for health care to partner with social service organizations to address SDH impacting health. This would provide opportunities for reimbursement of some Brenner FIT activities, in particular those related to food insecurity and nutrition education.

## Discussion

Community partnerships addressing pediatric obesity [38] typically extend treatment and prevention programs to community settings to increase access and availability and build community capacity; these partnerships are valuable and effective [15,16]. Given the impact of poverty and other SDH on health, obesity treatment programs benefit from engagement with community partners to support family health behavior change and address SDH with additional resources. Our vignettes describe “non-traditional’ approaches to community partnerships to support the health of individual children and their families, particularly those burdened by obesity, poverty, and lack of access to care.” Trust built with community organizations – not a quantifiable metric – is nonetheless clearly seen in the varied programs described here, many of which are now sustained through external resources.

These collaborations are distinct from coalitions or interventions. Community coalitions aim to improve an entire community’s health, whereas community-clinic partnerships aim to improve the health of individuals or distinct groups, aka patients. In these partnerships, the community’s capacity can be augmented to address obesity; on the other hand, clinical programs can be supported, extended, and deepened by community partners. These are also distinct from community-based interventions, where the treatment is provided in a local setting, versus a partnership addressing the issue together.

Community engagement provides a framework for community partners and obesity clinicians to collaborate [10], termed “Rules of Engagement.” These are principles of community engagement that assist in building effective relationships. Using these principles to address obesity treatment barriers and challenges to care, Brenner FIT seeks to:

- *Enlist new resources and allies:* We collaborate with many diverse local community groups. These include Spanish-speaking mental health providers, hunger relief organizations, water safety educators, bicycle advocates, and parenting support programs.
- *Build trust:* In-person meetings are held in community settings as a first step to build trust. Both community organizations and Brenner FIT benefit from these partnerships. Community partners often would request additional programming or would identify ongoing needs of their organization. Examples include Brenner FIT providing continuing education to members of the County Health Department and leading cooking classes for the CSA programs.
- *Create better communication:* Exchanging email addresses, individual office phone numbers, and other sources of communication (e.g., mobile telephone numbers for SMS messaging) are effective means to facilitate connections. Brenner FIT purchased a centralized mobile phone that rotates among team members, so that community organizations can more easily reach them and keep in touch.
- *Improve overall health outcomes and develop lasting collaborations:* While some collaborations have been short term (e.g., 2–3 years for the for-profit produce delivery company), others are ongoing (e.g., the YMCA). Furthermore, relationships have evolved depending on the partner and situation. Several successful grants resulted from collaborations; these have increased community capacity to assess outcomes and evolve programs in new directions.

Based on our experience, we have deemed several lessons worthy of sharing:

- *Collaborations and partnerships take time*. Learning each other’s mission and vision, areas of expertise, and service populations is not a swift process.
- *Learning is mutual*. Brenner FIT has had the opportunity to educate partners on weight bias, preferred terminologies, dangers of fad diets, and contributors to the obesity epidemic. In turn, Brenner FIT learned the intricacies of culinary instruction, stressors associated with children having unauthorized parents, barriers to Latinx mental health services, the importance of water safety education, and the culinary challenges of cooking seasonally with donated produce.
- *Communication styles vary greatly outside of clinical settings*. Many community partners do not email as frequently as academics, instead preferring to talk by telephone or in-person. Telephone calls or emails are sometimes returned weeks later, even when community partners were eager to collaborate. After initial contact, meeting potential collaborators in the community or place of their choosing resulted in improved future communication.
- *Relationships are the first step*. Partnerships change over time, which could reflect changes in funding, interest, and availability. Sometimes an appropriate goal is just to establish a working relationship as a foundation for future projects.
- *Be prepared to provide service to the community*. Clinicians may sometimes consider partnerships as part of their “work” in serving patients. But many community partners, especially non-profits, place a high value on sharing knowledge and resources.
- *Capacity building should be at the forefront*. When Brenner FIT’s ability to provide nutrition and parenting education to partners was limited (due to demand and staff availability), a curriculum was developed and shared with a few partners. Staff in those groups were trained in the curriculum, shadowed Brenner FIT clinicians, and could use its educational materials and teaching kitchen. This approach strengthened the community-clinic collaboration and increased partners’ capacity to deliver evidence-based educational material in community settings.

Developing effective partnerships with community organizations can offer additional resources to families struggling with weight issues and help to overcome barriers to care among SDH – such as food insecurity, lack of transportation, and mental health support. It is our hope that collaborations such as those our clinic has developed can be examples for other programs to adapt as they grow their efforts to address deep-seated issues contributing to poor health in our communities. In the future, a more holistic approach to evaluating outcomes is needed to assess direct and indirect impact of these interventions and collaborations; Armstrong and Skinner note the need for a deeper definition of “clinically significant” change recognizes that positive behavior change may precede change in weight status [39]. Similarly, we can assess the impact on overall health that addressing SDH can bring, such as improved quality of life, decreased stress, and improved physical activity levels. Program evaluation can include these broader measures to capture the full impact of addressing SDH during weight management.
